# Evaluation of a rapid diagnostic test for *Schistosoma mansoni* infection based on the detection of circulating cathodic antigen in urine in Central Sudan

**DOI:** 10.1371/journal.pntd.0008313

**Published:** 2020-06-19

**Authors:** Mohamed M. Elbasheir, Ibrahim A. Karti, Elwaleed M. Elamin

**Affiliations:** 1 Department of Parasitology & Medical Entomology, Faculty of Medical Laboratory Sciences, Alzaiem Alazhari University, Khartoum State, Sudan; 2 Department of Preventive Medicine, Omdurman Military Hospital, Khartoum State, Sudan; 3 Department of Histopathology & Cytology, Faculty of Medical Laboratory Sciences, Alzaiem Alazhari University, Khartoum State, Sudan; Federal University of Agriculture Abeokuta, NIGERIA

## Abstract

**Background:**

The Kato-Katz thick smear is the standard test for the diagnosis of *Schistosoma mansoni* infection, but the sensitivity of this technique is low. As an alternative, (CCA) strip test has been evaluated with the conclusion that it may replace the Kato-Katz method in areas where prevalences are moderate or high. Therefore, this study was undertaken to evaluate the performance of the CCA strip test in the diagnosis and monitoring of *S*. *mansoni* infection in Sudan.

**Methodology:**

489 stool and urine samples were collected from school children in endemic area of Sudan to determine the validity of CCA strip test based on duplicate Kato-Katz thick smear technique. Additional, 118 samples from known non schistosome-endemic area were collected to assess the CCA cross reactivity with other pathogens rather than schistosomiasis. The stability of CCA in urine samples was determined by consecutive examination of 40 positive CCA urine samples. 81 samples were used to evaluate the CCA strip test for the assessment of cure one week, three weeks and six weeks post Praziquantel treatment.

**Principal findings:**

Assuming parasitological test results as the gold standard, the sensitivity, specificity, positive predictive and negative predictive values of the CCA test were 96%, 85.4%, 78.5% and 97.5% respectively. There was no cross reactivity with other pathogens. The CCA strip test showed high accuracy in monitoring of treatment 93.8% and 100% after three and six weeks of administration of Praziquantel respectively. The stability of the CCA for long time in the urine revealed a safety transportation and shipment of the samples whenever it demanded.

**Conclusion/Significance:**

The uses of urine CCA strip test in the field would provide more accurate information on the epidemiology and monitoring of the *Schistosoma mansoni* infection in endemic areas of schistosomiasis than the conventional parasitological method. Moreover, The stability of CCA in urine samples confirms a safety transportation period of the samples whenever it required.

## Author summary

A newly developed one-step reagent strip test for detection of *S*. *mansoni* circulating cathodic antigen (CCA) in urine was evaluated in different countries and claimed that it has high sensitivity as well as specificity for the epidemiological study of schistosomiasis. However, it is of practical importance to evaluate the effectiveness of such a new technique in several schistosomiasis-endemic areas prior to its application for routine diagnosis or epidemiological study of the disease. Therefore, the present study aimed to evaluate this new method in order to be adopted and applied in the Sudan to improve the diagnosis and to assist in the control of schistosomiasis. 489 school children in central Sudan were examined for stool and urine using Kato-Katz technique and CCA strip test for *S*. *mansoni* infection. The sensitivity, specificity, positive predictive and negative predictive values of the CCA test were 96%, 85.4%, 78.5% and 97.5% respectively. The CCA strip test also showed high accuracy in monitoring of treatment 93.8% and 100% after three and six weeks of administration of Praziquantel respectively. Therefore, the uses of urine CCA strip test in the field would provide more accurate information on the epidemiology and monitoring of the *Schistosoma mansoni* infection in endemic areas of schistosomiasis. Additional assessment is recommended in areas with lower schistosomiasis prevalence and intensity levels.

## Introduction

Intestinal schistosomiasis is one of the major public health problems in Sudan [1]. The disease is highly endemic in the central Sudan, an area where the vast majority of the irrigating schemes were constructed. The overall infections are varied according to area [1]. The sugar cane schemes showed the higher rate of infection, 24%, 25.6%and 27% in Kenana, White Nile, and Sennar schemes respectively[2,3,4]. Studies carried out in Sennar states showed different rates of infections ranging from 5.0% to 47% in different villages of the states with overall prevalence of *S*. *mansoni* was found to be 27% [4]. Epidemiological assessment of the disease is usually based on detection of eggs in faeces using Kato-Katz technique. The sensitivity of this method is diminished when the prevalence and intensity are low, as well as in chronic infections and in post treatment situations. In addition, microscopic techniques are not sufficient for the diagnosis of recent infections in which worms have not started to produce eggs [5].

In epidemiological studies multiple stool examination is operationally demanding and people usually refused to provide repeated stool samples [6].

The existence of cross-reactivity with other helminthic infections and its low specificity after treatment, due to the delayed clearance of specific antibody levels, eliminates the use of immunological methods in both epidemiological studies and control programme [7].

Detection of circulating anodic and cathodic antigens in urine or serum of infected individuals using ELISA has been shown to be an alternative method that overcomes the disadvantages of detection of antibodies [8,9]. However, these techniques can not routinely be used in the field for community diagnosis because of field laboratory settings.

Recently, one of the antigen-ELISA’s, detecting CCA in urine, has been converted into a field-applicable reagent strip test. This test is easy, robust, applicable in the field, and highly sensitive and specific, in particular for diagnosis of schistosomiasis mansoni as shown in trials conducted in Tanzania, Kenya, Uganda, Ethiopia and South Sudan [10,11,12,13,14] respectively. However, It is of practical importance to evaluate the effectiveness of such a new technique in several schistosomiasis-endemic areas with variable intensities of infection prior to its application for routine diagnosis or epidemiological study of the disease. The present study aimed to evaluate the performance of the CCA strip test in the diagnosis and monitoring of *S*. *mansoni* infection in order to be adopted and applied in Sudan to improve the diagnosis and to assist in the control of schistosomiasis.

## Materials and methods

### Ethics statement

Permission of the study was obtained from the federal Ministry of Health (Ethical Committee, Protocol No. 21/2017) and local authorities of the Ministries of Health and Education of Sennar state. The aim of the study was discussed with head masters of the schools, informed consent was signed by the children’s parents and verbal assent been provided by participating children. All infected children determined by stool examination was treated with the appropriate dose of Biltricide praziquantel (40 mg/kg body weight).

### Study design and population

This is a longitudinal survey conducted in West Sennar villages (Sennar state) located about 275 km south of Khartoum state. This area is located beside sugar factory and surrounded with canals and creeks to irrigate the sugar cane scheme. Infection with intestinal schistosomiasis is dominant in this area with a prevalence rate of 27% [4]. The study area is comprised of four villages, each village’s populations range between 3400–4600 inhabitants with agriculture as the predominant way of living. No previous treatment of schistosomiasis had been administrated in the area for the last one year prior to this study. Bathing and swimming in the canals were the most frequent causes of schistosomiasis transmission [4].

Another selected groups of 118 individuals infected with other pathogens rather than *Schistosoma mansoni* attended to Khartoum Tropical Areas Hospital in Khartoum state were tested for *Schistosoma mansoni* infection in order to assess the CCA strip test for cross-reactivity with the common pathogens in the country.

### Sample collection and laboratory procedures

We recruited children from four different coeducational elementary schools using non-probability convenience sampling. We enrolled 489 schoolchildren, of whom 291 were male and 198 female during the period from February 2017 to March 2018. The children ranged between 5 and 15 years of age, with a median age of 12 years. Stool samples were subjected to Kato-Katz thick smears for the diagnosis of *S*. *mansoni* eggs. In addition, the other intestinal helminthes eggs were also identified. Urine samples were assessed with CCA strip test for the detection of *S*. *mansoni’*s circulating cathodic antigen.

Faecal specimens were processed using two thick smears of Kato-Katz technique [15] from one stool sample, with template for 41.7 mg of stool per slide, after which the slides were examined microscopically for *S*. *mansoni* eggs and other intestinal helminths.

The eggs were counted and the intensity of infection was expressed as eggs per gram of faeces (epg) for each subject and categorized as light, moderate and heavy infection [16].

Urine specimens were immediately tested using urine CCA strips according to the protocol supplied (Department of Parasitology, LUMC, The Netherlands). 25 μL of urine was added to a tube containing dried carbon conjugated antibody, along with 75 μL of buffer that was provided by the supplier. Test strips were inserted to the mixture of the urine and the buffer and allowed to develop for one hour. Strips were removed, allowed to dry, and read against standards provided by the manufacturer. In case the control bands did not develop, the test was considered invalid and the urine sample was retested with a new strip test. As the CCA strips were read, they were compared to the standards and scored as 0, 1, 2 or 3. A score of 0 indicated a negative result where the test line was weaker than the 100 standard, score 1 indicated that a test line was same as the 100 but weaker than control line, score 2 indicated a test line was strong as control line (1000 standard), and score 3 indicated that a test line was stronger than control line (10000) standard. Results were determined blindly by two technicians. Further details on the method were described by Shane et al.[11].

Cross-reactivity of the CCA strip test was evaluated by examination of selected groups whom were known positive for common frequent pathogens namely, *Hymenolepis nana* (n = 31), *Leishmania donovani* (n = 12), Hepatitis B virus (n = 09), *Entamoeba histolytica* (n = 7), *Giardia lamblia* (n = 11), *Plasmodium falciparum* (n = 14) and 34 patients with urinary tract infection. These groups were examined and diagnosed by senior technologists in Khartoum Tropical Areas Hospital. The selected groups also confirmed microscopically for *S*. *mansoni* infection by using triplicate Kato-Katz technique then subjected to CCA urine strip test evaluation.

In order to determine the stability of CCA in the urine samples forty CCA-positive urine samples were put first at room temperature for 24 hours, then the same forty samples were put in the refrigerator (4 up to 8 °C) for seven days and then for one year at -20 °C. The urine samples were subjected for consecutive examination by using CCA strip test and the results were recorded and compared to the initial results.

### Evaluation of post-treatment efficacy of CCA test strips

A 6-weeks survey after treatment was conducted. Overall, (175) individuals infected with *Schistosoma mansoni* (based on Kato-Katz method) were treated with Biltricide Praziquantel (40 mg/kg body weight).

81 individuals were successfully followed-up post-treatment and used for the evaluation of the effectiveness of the CCA strip test in monitoring the efficacy of praziquantel. Urine samples were subjected to CCA strip test for *S*. *mansoni* one week after treatment. Stool and urine samples collected 3 and 6 weeks after the administration of praziquantel were subjected to the same diagnostic tests as during the pretreatment; two thick smears of Kato-Katz technique and urine CCA strip test, respectively.

## Data analysis

Data were analysed using SPSS version 11.5 software (SPSS for Windows). The CCA reagent strip was evaluated in terms of sensitivity, specificity and positive predictive and negative predictive values. Microscopic examination of faecal samples using Kato-Katz technique was considered as the gold standard.

Individuals infected with other pathogens were used to assess the cross reactivity of the strip test. Stability of CCA in urine samples was determined by comparing of the test line intensity throughout the storage approaches with the initial band intensity at baseline.

## Results

### Validity of CCA strip test in the diagnosis of *S*. *mansoni* infection

Among the 489 stool samples examined with the duplicate Kato-Katz technique, 175 were found to be positive for *S*. *mansoni*. Using the CCA strip test, 168 of these cases were found positives while the remaining 7 cases showed negative results. Out of 314 urine samples from stool-negative cases examined by the CCA strip test, 46 samples were found positive, constituting 96% sensitivity of the reagent strip test while its specificity was 85.4%. The positive predictive value and negative predictive value were 78.5% and 97.5% respectively (Table 1). Interestingly, those 46 CCA positives cases were followed up by Kato-Katz examination, single time every week and all are found to be microscopically positive within seven weeks.

**Table 1:** Comparison of Kato-Katz method with CCA strip test in the diagnosis of *S*. *mansoni* infection.

Urine CCA strip Test	Stool examination by Kato-Katz	
	Positive	Negative
Positive	168	46
Negative	7	268
**Total**	**175**	**314**

### Prevalence of *S*. *mansoni* infection

Assuming the Kato-Katz method, the prevalence of *Schistosoma mansoni* infection in West Sennar villages was 35.8% while the CCA strip method revealed a prevalence rate of 43.8% (Table 2).

**Table 2:** Prevalence of *S*. *mansoni* infection in the study area by Kato-Katz and CCA strip methods.

Method	No. examined	Positive	Negative	Prevalence %
**Kato-Katz**	**489**	**175**	**314**	**35.8**
**CCA strip**	**489**	**214**	**275**	**43.8**

### Intensity of infection in individuals with *S*. *mansoni* infection.

As it shown in Table 3, out of the total 175 positive subjects by Kato-Katz 81 (46.3%), 69 (39.4%) and 25 (14.3%), were found to have light, moderate and heavy infections, respectively.

**Table 3:** Intensity of infection in individuals with *S*. *mansoni* infection determined by Kato-Katz method.

Intensity of infection by Kato-Katz			Total
1–99 eggs/gram faeces (light)	100–399 eggs/gram faeces (moderate)	≥ 400 eggs/gram faeces (heavy)	
**81 (46.3%)**	**69 (39.4%)**	**25 (14.3%)**	**175 (100%)**

### Determination of CCA strip test cross-reactivity

None of the selected groups of known infected with common pathogens were found to be positive by the CCA urine strip, showing no cross-reactivity with other pathogenic’s antigens.

### Stability of CCA in urine samples

The stability of CCA in urine samples was determined by consecutive examination of 40 positive CCA urine samples. The score of the test line of the CCA strip did not change when the tested urines stored for 24 hours at room temperature and for a period of 7 days at 4 up to 8 °C. In addition, no change was observed in the test line when the urine samples were kept for one year at -20 °C. These particular situations are the potential conditions for storing samples and allow examination whenever it demanded.

### Effect of treatment on CCA strip test results

**Fig 1:**
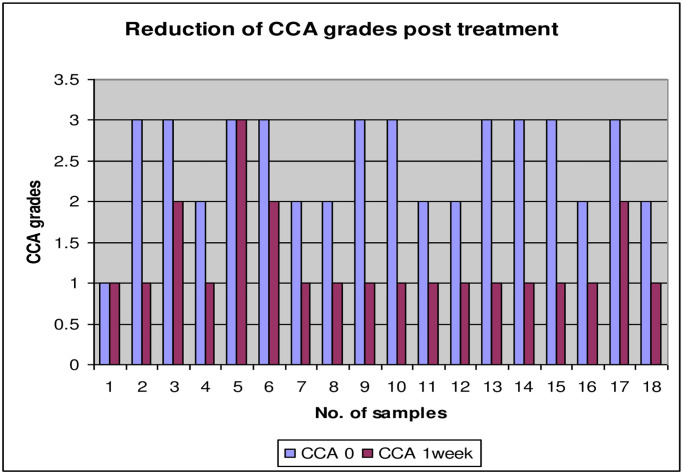
Reduction of CCA scores (grades) one week post-treatment.

**Fig 2:**
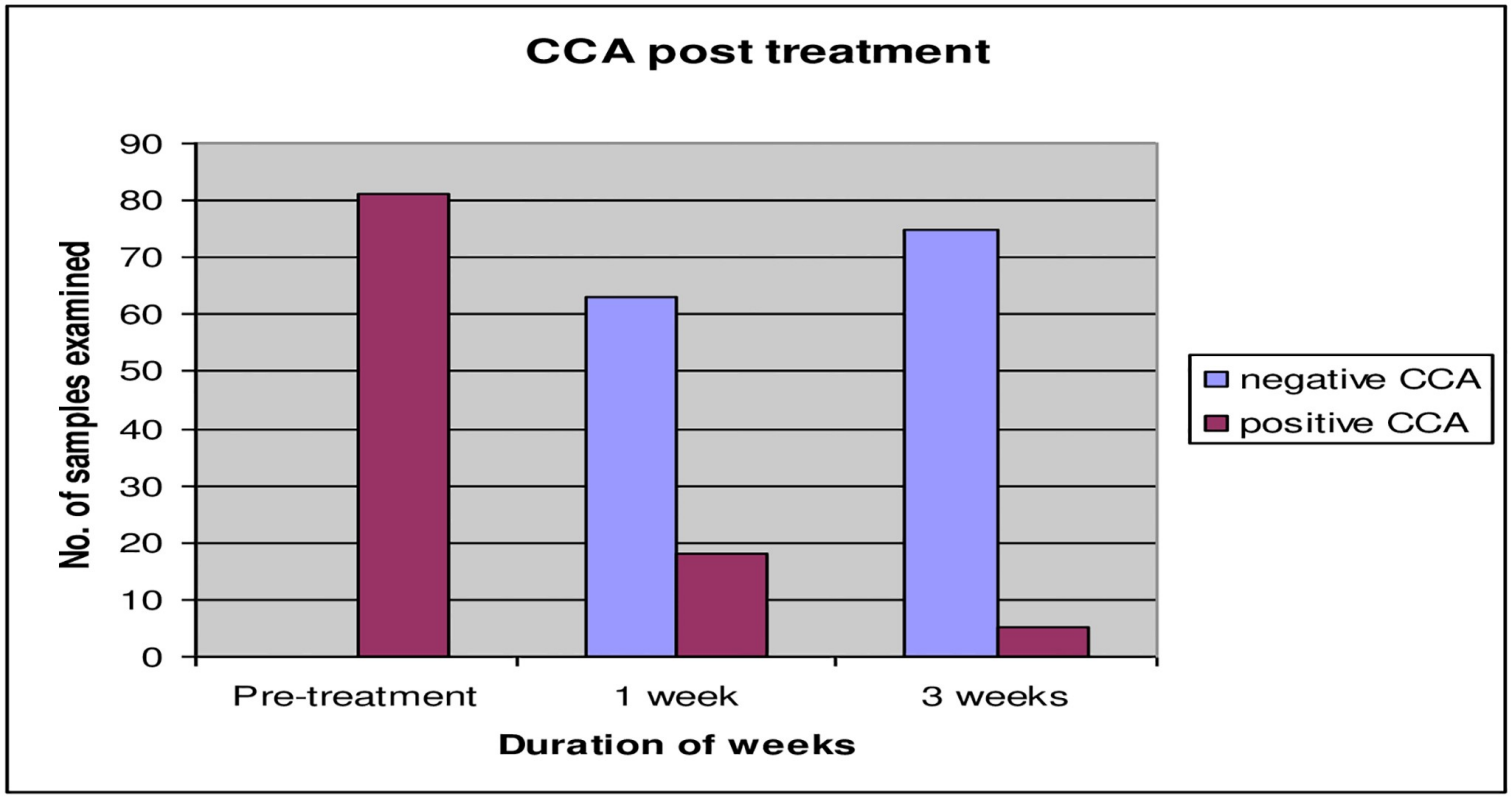
Effect of treatment on CCA strip test results within three weeks post-treatment.

After Praziquantel treatment, out of 81 positive CCA which were also positive microscopic, 63 individuals showed negative CCA after one week (77.8%) while among the remaining 18 positive CCA, 16 samples (88.9%) showed reduction in the scores (grades) of the strip test (Fig 1). Three weeks later, the 81 positive samples were re-examined using the two methods, only one sample was found to be positive for *S*. *mansoni* with light infection (secreted viable eggs) by microscopic and CCA strip test and 4 samples were found to be positive CCA with reduction to score 1, while 76 samples were negative result of CCA (93.8%) (Fig 2). Six weeks post treatment, all samples were negative by both methods. The CCA was completely disappeared (100%).

### Discussion

The present study was carried out in order to assess the accuracy of a recently developed reagent strip for the diagnosis of infection with *S*. *mansoni* by detecting CCA in urine under field condition. The results revealed that detection of CCA in urine using reagent strip test would gives an accurate information on the epidemiology and monitoring of *Schistosoma mansoni* infection than Kato Katz and can be used for detection of the infection even before the worms start to produce eggs, as previously reported by van Dam et al. [10]. In comparison to others, the sensitivity of the test was higher than that observed elsewhere [11,13,17,18,19]. The differences observed between the present and previous studies might be due to the difference in the prevalence of the disease. In addition, this can be attributed to the finding previously reported by van Dam et al.[10] that the test may has failed to identify light infections. Nevertheless, in the current study, although most of the infected subjects (46.3%) had light infections as determined by Kato-Katz method, only 4.0% showed negative CCA which was not in line with the previous observation. However, it agreed with the results suggested by van Lieshout et al. [20] who found that high sensitivity was achieved with the urine-CCA assay in low intensity areas. Specificity described in the present study was in agreement with the specificity reported previously, [13,10,11] while it was in contrast with other authors [19,17,18,21]. This disparity might probably be due to the false positive results which may occurred due to the effect of day-to-day variation of egg output in stool as previously observed, or the eggs produced were fewer to be detected by Kato-Katz examination, which is known to be insensitive [22]. The false positive results, also, may have occurred when the individuals have harboured young schistosomes that were pre-patent in their egg laying. The present study confirmed the latest explanation when the individuals with CCA-positive and egg-negative, were subjected to follow up for several weeks (up to seven weeks), eventually, all cases were shown to be microscopic positive suggested that the CCA strip method may detect the infection earlier even before the worms start to produce eggs. Another possible explanation of the lower specificity is the general inflammatory biomarkers excreted in the urine, possessing Lewis-X tri-saccharide epitopes, which might cross-react with the CCA strip to illicit a non specific result [23,24]. Indeed Ayele et al. [25] reported cross-reactivity in individuals with urinary tract infection (UTI). In contrast, the present study has not found a positive case by the CCA urine strip among urinary tract infected people or individuals infected by other pathogens. In this study, the positive predictive and high negative predictive values reported were in agreement with the results reported by other authors [17,26] but they were different from those results obtained elsewhere [18]. This variation in the predictive values with the other studies might be due to the relatively low observed sensitivity and specificity of the CCA and also might be due to the prevalence of the disease, which could affect the predictive values as previously reported [17]. Thus, the positive predictive value of the CCA strip test could be high in areas where the prevalence of *S*. *mansoni* is high, whereas it decreased in low prevalence areas. One of the known advantages of detecting circulating antigens in serum or urine from *S*. *mansoni* infected patients is the high effectiveness in assessment of the efficacy of treatment following Praziquantel therapy [27,28] which is in agreement with the results found in the current study. In the present study, the accuracy of CCA post treatment was 77.8% after one week and 93.8% after three weeks. Similarly, van Lieshout et al.[29]observed high reduction rate in CCA titre after one week and specificity of 92% three weeks after treatment. Six weeks after treatment, the current study showed a specificity of 90.3% of CCA which was similar to the results reported in other studies 95.8% [30] and a complete disappearance of CCA in urine sample100%[27]. Less specificity was found to be 75% [17]. In contrast, van Lieshout et al. [31] observed high level of CCA in urine from *S*. *mansoni* patients six weeks post treatment with the same dose of Praziquantel (40 mg/kg body weight). This is probably due to treatment failure as those patients were still found secreting viable eggs. The high cure rate after 3–6 weeks of administration of Praziquantel found in this study is similar to the cure rate that was found by other authors [32,33]. The present study described for the first time the stability of CCA in urine samples for 24 hours at room temperature, one week at refrigerator and for one year at deep freezing confirms a safety transportation and shipment of the samples whenever it required. In conclusion, the results of the current study indicate that the diagnosis of *S*. *mansoni* by detecting CCA in urine using a recently developed reagent strip test would gives an accurate information on the epidemiology and monitoring of *Schistosoma mansoni* infection and can be used for screening and mapping of the infection in both moderate and high endemic settings. Moreover, The stability of CCA in urine samples confirms a safety transportation period of the samples whenever it required. Additional assessment is recommended in areas with lower schistosomiasis prevalence and intensity levels.

## Supporting information

S1 Fig Checklist STARD-Checklist. (DOCX)

S2 Fig Flow Diagram STARD Flow Diagram. (JPG)
