# Supplementary material for: Evaluation of a rapid diagnostic test for Schistosoma mansoni infection based on the detection of circulating cathodic antigen in urine in Central Sudan
Source: PLoS Negl Trop Dis. 2020 Jun 19;14(6):e0008313. doi: 10.1371/journal.pntd.0008313 (PMC7360052; doi:10.1371/journal.pntd.0008313)
Supplement: S1 Fig — (DOCX) [file pntd.0008313.s001.docx]

|  | **Section & Topic** | **No** | **Item** | **Reported on page #** |
| --- | --- | --- | --- | --- |
|  |  |  |  |  |
|  | **TITLE OR ABSTRACT** |  |  |  |
|  |  | **1** | Identification as a study of diagnostic accuracy using at least one measure of accuracy  (such as sensitivity, specificity, predictive values, or AUC) | Abstract, Principal Findings, paragraph 1 Page 2 |
|  | **ABSTRACT** |  |  |  |
|  |  | **2** | Structured summary of study design, methods, results, and conclusions  (for specific guidance, see STARD for Abstracts) | Abstract, paragraph 2 Page 1, paragraph 1,2 Page 2 |
|  | **INTRODUCTION** |  |  |  |
|  |  | **3** | Scientific and clinical background, including the intended use and clinical role of the index test | Introduction, paragraph 1, Page 3 |
|  |  | **4** | Study objectives and hypotheses | Introduction, paragraph 4  Page 4 |
|  | **METHODS** |  |  |  |
|  | *Study design* | **5** | Whether data collection was planned before the index test and reference standard  were performed (prospective study) or after (retrospective study) | Methods, paragraph 3  Page 4, paragraph 1  Page 5, |
|  | *Participants* | **6** | Eligibility criteria | N / A |
|  |  | **7** | On what basis potentially eligible participants were identified  (such as symptoms, results from previous tests, inclusion in registry) | N / A |
|  |  | **8** | Where and when potentially eligible participants were identified (setting, location and dates) | Methods, paragraph 2  Page 4 |
|  |  | **9** | Whether participants formed a consecutive, random or convenience series | Methods, paragraph 3  Page 4 |
|  | *Test methods* | **10a** | Index test, in sufficient detail to allow replication | Methods, paragraph 2  Page 5 |
|  |  | **10b** | Reference standard, in sufficient detail to allow replication | Methods, paragraph 1  Page 5 |
|  |  | **11** | Rationale for choosing the reference standard (if alternatives exist) | N /A |
|  |  | **12a** | Definition of and rationale for test positivity cut-offs or result categories  of the index test, distinguishing pre-specified from exploratory | Methods, paragraph 2  Page 5 |
|  |  | **12b** | Definition of and rationale for test positivity cut-offs or result categories  of the reference standard, distinguishing pre-specified from exploratory | Methods, paragraph 1  Page 5 |
|  |  | **13a** | Whether clinical information and reference standard results were available  to the performers/readers of the index test | Methods, paragraph 1  Page 5 |
|  |  | **13b** | Whether clinical information and index test results were available  to the assessors of the reference standard | Methods, paragraph 2  Page 5 |
|  | *Analysis* | **14** | Methods for estimating or comparing measures of diagnostic accuracy | Methods, paragraph 2  Page 6 |
|  |  | **15** | How indeterminate index test or reference standard results were handled | N/A |
|  |  | **16** | How missing data on the index test and reference standard were handled | N/A |
|  |  | **17** | Any analyses of variability in diagnostic accuracy, distinguishing pre-specified from exploratory | Methods, paragraph 2  Page 6 |
|  |  | **18** | Intended sample size and how it was determined | Methods, paragraph 3  Page 4, paragraph 1  Page 6 |
|  | **RESULTS** |  |  |  |
|  | *Participants* | **19** | Flow of participants, using a diagram | N /A |
|  |  | **20** | Baseline demographic and clinical characteristics of participants | N/A |
|  |  | **21a** | Distribution of severity of disease in those with the target condition | Result, paragraph 1,2, Page 8 |
|  |  | **21b** | Distribution of alternative diagnoses in those without the target condition | Result, paragraph 2, Page 7 |
|  |  | **22** | Time interval and any clinical interventions between index test and reference standard | Result, paragraph 2,3 Page 9 |
|  | *Test results* | **23** | Cross tabulation of the index test results (or their distribution)  by the results of the reference standard | Result, paragraph 1, Page 7 |
|  |  | **24** | Estimates of diagnostic accuracy and their precision (such as 95% confidence intervals) | Result, paragraph 3, Page 6 |
|  |  | **25** | Any adverse events from performing the index test or the reference standard | N/A |
|  | **DISCUSSION** |  |  |  |
|  |  | **26** | Study limitations, including sources of potential bias, statistical uncertainty, and general- isability | N/A |
|  |  | **27** | Implications for practice, including the intended use and clinical role of the index test | Discussion paragraph 3, Page 11, 12 |
|  | **OTHER INFORMATION** |  |  |  |
|  |  | **28** | Registration number and name of registry | N/A |
|  |  | **29** | Where the full study protocol can be accessed | N/A |
|  |  | **30** | Sources of funding and other support; role of funders | Acknowledgments  Page 12 |
|  |  |  |  |  |
