# Supplementary figures and images for: Evaluation of a rapid diagnostic test for Schistosoma mansoni infection based on the detection of circulating cathodic antigen in urine in Central Sudan
Source: PLoS Negl Trop Dis. 2020 Jun 19;14(6):e0008313. doi: 10.1371/journal.pntd.0008313 (PMC7360052; doi:10.1371/journal.pntd.0008313)

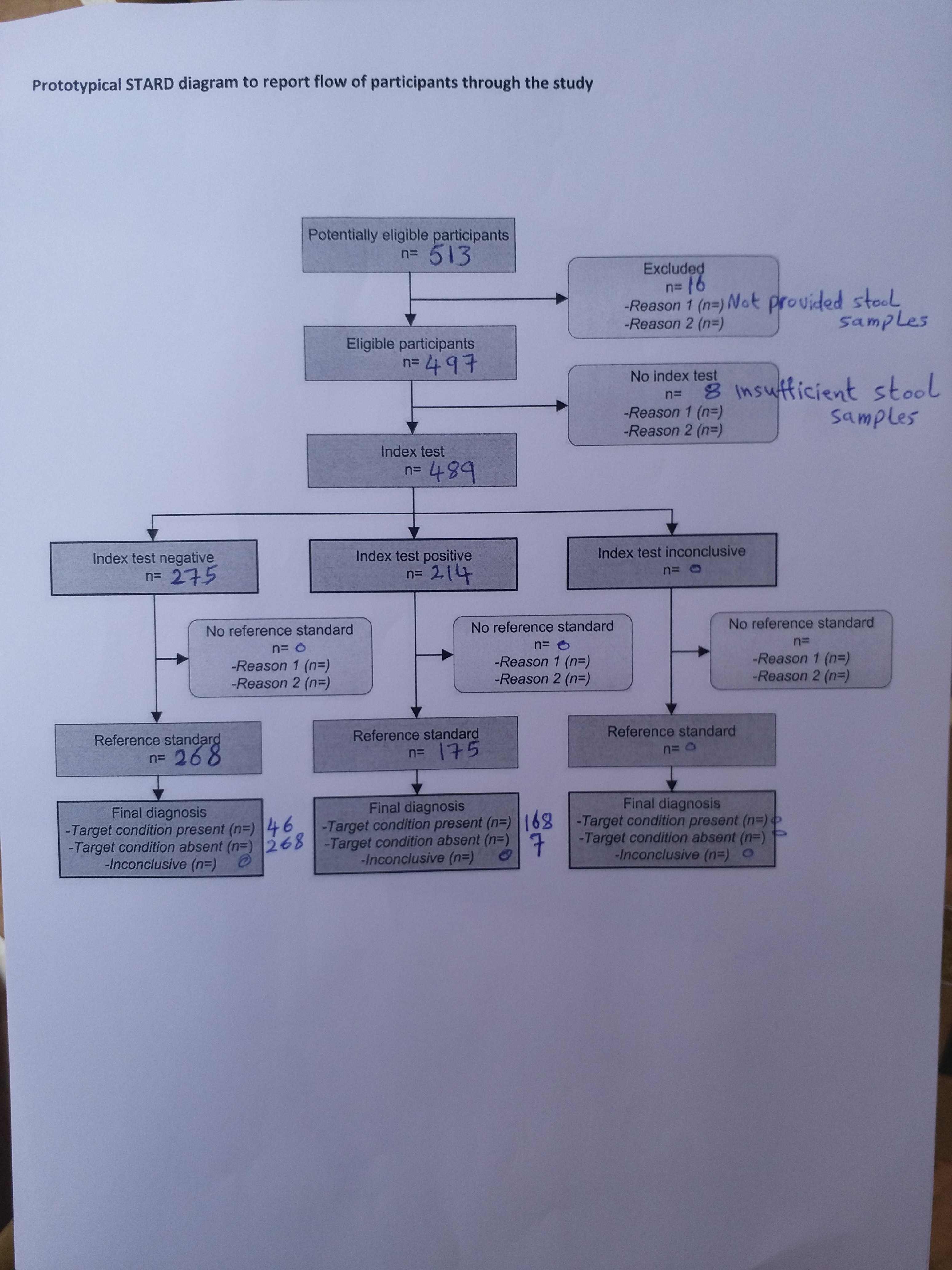

Supplement: S2 Fig — (JPG) [file pntd.0008313.s002.jpg]
